# Retraction: Overexpression of Wnt7a enhances radiosensitivity of non-small-cell lung cancer via the Wnt/JNK pathway

**DOI:** 10.1242/bio.058582

**Published:** 2022-06-28

**Authors:** Pingping Ai, Xianhua Xu, Shijie Xu, Zhixia Wei, Shun Tan, Junzhe Li

This notice updates and replaces the Expression of Concern (doi:10.1242/bio.054924).

The journal is retracting *Biology Open* (2020) **9**, bio050575 (doi:10.1242/bio.050575) because of concerns with the reliability of the data.

After issues were raised by the Editor-in-chief about background duplications in the blots in Figs 3 and 5, and possible duplicated FACS data in Fig. 2, the authors were asked to supply all their original data. Unfortunately, the authors could not provide the original data, despite the paper being published so recently. The authors re-analysed their samples and supplied cropped replicate blots and analyses, stating that these results support the conclusions of the paper.

The journal concluded that without the original data for the published figures, we have no option but to retract this paper. This course of action follows the advice set out by COPE (Committee on Publication Ethics), of which Biology Open is a member.

The authors did not respond to say whether they agree with this retraction.

